# SARS-COV-2 vaccine responses in renal patient populations

**DOI:** 10.1186/s12882-022-02792-w

**Published:** 2022-05-31

**Authors:** Rona M. Smith, Daniel J. Cooper, Rainer Doffinger, Hannah Stacey, Abdulrahman Al-Mohammad, Ian Goodfellow, Stephen Baker, Sara Lear, Myra Hosmilo, Nicholas Pritchard, Nicholas Torpey, David Jayne, Vivien Yiu, Anil Chalisey, Jacinta Lee, Enric Vilnar, Chee Kay Cheung, Rachel B. Jones

**Affiliations:** 1 Department of Medicine, University of Cambridge, Cambridge, UK; 2 Cambridge University Hospitals NHS Foundation Trust, Cambridge, UK; 3 Department of Renal Medicine, Addenbrooke’s Hospital, Hills Road, Box 118, Cambridge, CB2 0QQ UK; 4 Division of Virology, Department of Pathology, University of Cambridge, Cambridge, UK; 5 West Suffolk Hospital NHS Foundation Trust, Bury St Edmunds, UK; 6 East and North Hertfordshire NHS Trust, Stevenage, UK; 7 Department of Cardiovascular Sciences, University of Leicester, Leicester, UK; 8 University Hospitals of Leicester NHS Trust, Leicester, UK

**Keywords:** Vaccine, SARS-CoV-2, Immunosuppression, Rituximab, Mycophenolate, Transplant, Dialysis, Autoimmune, Antibody

## Abstract

**Background:**

Dialysis patients and immunosuppressed renal patients are at increased risk of COVID-19 and were excluded from vaccine trials. We conducted a prospective multicentre study to assess SARS-CoV-2 vaccine antibody responses in dialysis patients and renal transplant recipients, and patients receiving immunosuppression for autoimmune disease.

**Methods:**

Patients were recruited from three UK centres (ethics:20/EM/0180) and compared to healthy controls (ethics:17/EE/0025). SARS-CoV-2 IgG antibodies to spike protein were measured using a multiplex Luminex assay, after first and second doses of Pfizer BioNTech BNT162b2(Pfizer) or Oxford-AstraZeneca ChAdOx1nCoV-19(AZ) vaccine.

**Results:**

Six hundred ninety-two patients were included (260 dialysis, 209 transplant, 223 autoimmune disease (prior rituximab 128(57%)) and 144 healthy controls. 299(43%) patients received Pfizer vaccine and 379(55%) received AZ. Following two vaccine doses, positive responses occurred in 96% dialysis, 52% transplant, 70% autoimmune patients and 100% of healthy controls. In dialysis patients, higher antibody responses were observed with the Pfizer vaccination. Predictors of poor antibody response were triple immunosuppression (adjusted odds ratio [aOR]0.016;95%CI0.002–0.13;*p* < 0.001) and mycophenolate mofetil (MMF) (aOR0.2;95%CI 0.1–0.42;*p* < 0.001) in transplant patients; rituximab within 12 months in autoimmune patients (aOR0.29;95%CI 0.008–0.096;*p* < 0.001) and patients receiving immunosuppression with eGFR 15-29 ml/min (aOR0.031;95%CI 0.11–0.84;*p* = 0.021). Lower antibody responses were associated with a higher chance of a breakthrough infection.

**Conclusions:**

Amongst dialysis, kidney transplant and autoimmune populations SARS-CoV-2 vaccine antibody responses are reduced compared to healthy controls. A reduced response to vaccination was associated with rituximab, MMF, triple immunosuppression CKD stage 4. Vaccine responses increased after the second dose, suggesting low-responder groups should be prioritised for repeated vaccination. Greater antibody responses were observed with the mRNA Pfizer vaccine compared to adenovirus AZ vaccine in dialysis patients suggesting that Pfizer SARS-CoV-2 vaccine should be the preferred vaccine choice in this sub-group.

## Introduction

The renal patient population is diverse, including individuals requiring renal replacement therapy in the form of dialysis, and those receiving immunosuppression as a result of renal transplantation or for autoimmune diseases, such as vasculitis, systemic lupus erythematosus (SLE) or primary glomerulonephritis (GN). SARS-CoV-2 infection is associated with high morbidity and mortality in these complex patients [1]. The vulnerability of these individuals led to public health advice for them to adopt extended protective self-isolation measures early in the pandemic, and they were prioritised for vaccination against SARS-CoV-2. In the UK, most are being offered third doses of SARS CoV-2 vaccines in the autumn of 2021.

Vaccine trials have been successful in the general population and have shown that some vaccines are up to 95% effective at preventing symptomatic infection [2, 3]. However, patients with end stage kidney disease and those receiving immunosuppressive medications were largely excluded from the initial SARS-CoV-2 vaccine trials. Historically, vaccination against other infections results in suboptimal responses in these patients [4–6]. Vaccines against SARS-CoV-2 approved for use in the UK include the mRNA vaccines Pfizer BioNTech BNT162b2 (Pfizer) and Moderna mRNA-1273,; and the Adenovirus vector vaccines Oxford-AstraZeneca ChAdOx1 nCoV-19 (AZ) and Janssen Ad26.COV2-S.

Several groups have now reported on the immunogenicity of SARS-CoV-2 vaccines in immunocompromised patients. The serologic response to mRNA vaccines in patients receiving dialysis has been encouraging. One study, which included 1256 dialysis patients, reported seroconversion rates of 85–95% after the second dose; comparable to healthy controls [7]. However, antibody titres are lower when compared with healthy controls despite high seroconversion rates [8]. Most studies have assessed the effectiveness of mRNA vaccines and few report the neutralising capacity of antibodies, but a UK multi-centre cohort study of 178 haemodialysis patients revealed that infection-naïve patients had a markedly reduced neutralising antibody response to the AZ vaccine compared to the Pfizer vaccine, although the difference was reduced in individuals that experienced an infection prior to or vaccination [9]. Risk factors for reduced immunogenic response in dialysis patients included older age, co-morbidities and dialysis vintage.

In contrast, antibody production after mRNA vaccination is poor in individuals with a renal transplant with less than 60% generating protective level antibodies after the second dose [9, 10]. Comparison of the Pfizer and AZ vaccines in kidney transplant patients reported enhanced humoral responses with the Pfizer vaccine, however T-cell responses to both vaccines were attenuated when compared to healthy controls [11]. A reduced antibody response was associated with older age, more recent transplantation and anti-metabolite drugs, such as mycophenolate mofetil (MMF).

Less data is available on patients with autoimmune disease, and disease heterogeneity makes interpretation more challenging. The OCTAVE trial is assessing SARS-CoV-2 vaccine responses (to both Pfizer and AZ vaccines) in patients with chronic immune-mediated diseases on immunosuppression including individuals with inflammatory arthritis, ANCA-associated vasculitis, inflammatory bowel disease, hepatic disease and malignancy [12]. Preliminary data indicates that although 89% of patients seroconverted 4 weeks after the second vaccine dose, 40% generated lower levels of antibodies compared to healthy subjects. 72% of patients with ANCA-associated vasculitis (*N* = 30), who had all received rituximab, did not generate detectable antibodies, although T cell responses in all sub-groups were similar to those in healthy individuals. In addition to the poor antibody response following rituximab (particularly if rituximab has been administered within 6 months prior to the vaccine), glucocorticoids and methotrexate have also been associated with reduced antibody levels [13–16].

We conducted a prospective cohort study to assess differences and predictors of SARS-CoV-2 antibody responses following vaccination in three immunocompromised renal populations; dialysis, renal transplant and patients receiving immunosuppression for multisystem autoimmune disease. The use of the same assay across groups allowed direct comparison across renal patient populations as well as with healthy controls. Additionally, the majority of patients recruited to this study received the AZ vaccine, therefore addressing the relative paucity of data on responses to adenovirus vector vaccines in the existing literature. Our cohort includes over 100 individuals who have received the B-cell depletion therapy with rituximab, a population of particular interest.

## Methods

This prospective observational cohort study included patients recruited from the Departments of Nephrology, at Cambridge University Hospitals NHS Foundation Trust, East and North Hertfordshire NHS Trust and University Hospitals of Leicester NHS Trust in the UK (ethics reference: 20/EM/0180). Healthy controls were recruited at Cambridge University Hospitals NHS Foundation Trust (ethics reference: 17/EE/0025). Patients receiving dialysis or immunosuppression following either renal transplantation or for autoimmune renal disease were eligible. Patients receiving IVIg or plasma exchange were excluded to avoid potential confounders of vaccine response. Blood sampling was performed approximately three monthly for up to 18 months. Flexibility in the timing of blood samples was permitted to align sampling with 4–6 weeks post COVID vaccine administration during the study, as well as (wherever possible) aligning with routine clinical tests and access to blood testing facilities. Clinical data were collected from electronic medical records and patient interviews and included baseline demographics, changes to immunosuppressive medication over time and data on episodes of SARS-CoV-2 infection. SARS-CoV-2 IgG antibodies to spike protein (wild type Wuhan variant) were measured using a UKAS accredited multiplex Luminex assay following the first and second doses of either Pfizer BioNTech BNT162b2 (Pfizer) or Oxford-AstraZeneca ChAdOx1 nCoV-19 (AZ) vaccine. Spike antibody MFI titres greater than 1896 were considered positive, with a sensitivity of 92% and specificity of 99% as determined by ROC analysis, nucleocapsid antibody titres of > 6104 were considered positive for previous natural infection [17, 18]. Statistical analyses were performed using Stata version 14. Between group differences in proportional response to vaccine were compared using chi-squared test. Median spike antibody titres were compared using Wilcoxon rank-sum test. Multivariate logistic regression modelling was used to evaluate the odds of vaccine response.

## Results

### Patient characteristics and baseline demographics

Results on 692 patients are reported (260 dialysis; 209 transplant and 223 with autoimmune disease) between 21 January 2021 and 6th August 2021. Samples after the first vaccine dose were available for 234 (90%) dialysis, 180 (86%) transplant and 152 (73%) autoimmune disease patients and after the second dose for 236 (91%), 185 (89%) and 201 (90%) patients respectively. Median age was 62 (range 19–95) years. Diabetes was the cause of end stage renal failure in 59 (23%) of patients on haemodialysis. For those individuals not on dialysis, median eGFR was 62 ml/min (IQR: 42–90). Prior PCR confirmed SARS-CoV-2 infection was documented in 39 individuals (6%) (Table 1). A nucleocapsid titre suggestive of prior SARS-CoV-2 natural infection was seen in 50 individuals (7%). Healthy controls (*n* = 144) with s-antibody titres measured at 28 days post-second dose of vaccine were used for comparison. Median age was 40 (range 20–73) years; 32 (27%) were male, and all healthy controls had received two doses of the Pfizer vaccine (Pfizer BioNTech BNT162b2).

**Table 1:** Demographics according to patient group; dialysis, renal transplant or autoimmune disease

	Dialysis ***N*** = 260	Transplant ***N*** = 209	Autoimmune ***N*** = 223
Age (years) (median (range))	71 (20–95)	57 (23–78)	59 (19–88)
Sex (male) (Number (%))	149 (63)	108 (58)	90 (47)
Vaccine type (Number (%))			
- Pfizer	159 (61)	140 (67)	80 (36)
- AstraZeneca	92 (36)	69 (33)	138 (62)
- Unknown	9 (3)	0 (0)	5 (2)
Days between first and second vaccine doses (median (IQR))			
- Pfizer	77 (21–150)	77 (21–100)	76 (22–91)
- AstraZeneca	77 (25–144)	77 (35–102)	77 (27–135)
PCR proven prior Covid-19 infection (Number(%))	20 (8)	14 (7)	5 (2)
Samples after 1st vaccine dose (Number(%))	234 (90)	180 (86)	152 (73)
- Days after vaccination (median (IQR))	30 (18–88)	28 (14–75)	38 (21–118)
Samples after 2nd vaccine dose (Number(%))	236 (91)	185 (89)	201 (90)
- Days after vaccination (median (IQR))	30 (12–119)	30 (11–94)	33 (19–75)
S antibody titre after first vaccine dose (IU)	7450 (2940–19,457)	556 (189–3936)	1388 (157–11,714)
- Number(%) positive	189–234 (81)	61/180 (34)	69/152 (45)
S antibody titre after second vaccine dose (IU)	30,807 (26593–31,914)	4285 (295–26,230)	18,673 (1127–30,470)
- Number(%) positive	227/236 (96)	104/185 (56)	140/201 (70)

Dialysis patients were recruited from Cambridge (*n* = 217) and East and North Hertfordshire (*n* = 43). Data are reported at the time of most recent sampling. Antibody MFI titres greater than 1896 are considered positive

The median time from renal transplantation was 1264 (IQR 355–3788) days. 29 (14%) patients had received a combined kidney and pancreas transplant. 68 (33%) patients were on 3 agents for immunosuppression – most commonly low dose prednisolone, a calcineurin inhibitor (tacrolimus) and anti-metabolite (mycophenolic acid [MMF] or azathioprine). 130 (62%) were receiving MMF (Table 2). In the autoimmune population, disease subgroups were classified as: ANCA-associated vasculitis (AAV) 133 (60%); systemic lupus erythematosus (SLE) 27 (12%); large vessel vasculitis 11 (5%) and other 52 (24%). One hundred twenty-eight patients had received rituximab, a median of 162 (IQR 110–275) days prior to vaccination (Table 3).

**Table 2:** Detailed characteristics of the transplant group

	***N*** = 209
Time since most recent transplant (days) (median (IQR))	1264 (355–3788)
Cause of ESKD (Number (%))	
- Diabetic nephropathy	46 (22)
- Renovascular disease	7 (3)
- Glomerulonephritis	47 (23)
- Unknown	30 (14)
- Other	79 (38)
Type of transplant (Number (%))	
- Kidney	178 (85)
- Simultaneous kidney pancreas	29 (14)
- Other	3 (1)
Previous failed transplant (Number (%))	25 (12)
GFR (ml/min/m^2^) (median (IQR))	49 (34–90)
Concurrent immunosuppression^a^ (Number (%))	
- 3 agents	68 (33)
- 2 agents	109 (52)
- 1 agent	31 (15)
Mycophenolate mofetil (Number (%))^b^	130 (62)
- Daily dose (mg) (median (IQR))	1000 (500–1000)
Prednisolone (Number (%))	118 (56)
- Total daily dose (mg) (median (IQR))	3 (0–5)
Tacrolimus (Number (%))	205 (98)

All transplant patients were recruited from Cambridge. Data are reported at the time of most recent sampling. Basiliximab induction was used for low/medium risk kidney transplant recipients, alemtuzumab induction was used for high risk kidney and simultaneous pancreas kidney transplant recipients. ^a^Concurrent immunosuppression includes glucocorticoids. ^b^Mycophenolate mofetil (MMF) number includes all patients receiving any mycophenolic acid (MPA) preparation; doses were converted to the equivalent MMF doses for the purpose of understanding relative MPA dosing. Fourteen patients had prior rituximab exposure; 10 within the last 5 years; 5 for post transplant lymphproliferative disorder and 4 for autoimmune disease. Thirty-four received alemtuzumab induction

**Table 3:** Detailed characteristics of the autoimmune disease group

	***N*** = 223
Diagnosis (Number (%))	
- ANCA-associated vasculitis	133 (60)
- Large vessel vasculitis	11 (5)
- Systemic lupus erythematosus	27 (12)
- Behcet’s disease	17 (8)
- Other^a^	25 (16)
GFR (ml/min/m^2^) (median (IQR))	84 (56–90)
Rituximab (Number (%))	
- Received within 6 months	70 (31)
- Received within 12 months	108 (48)
- Received within 5 years	128 (57)
Days since last rituximab dose (median (IQR)	162 (110–275)
IV cyclophosphamide (Number (%))	
- Received within 6 months	76 (34)
- Received within 12 months	103 (46)
Days since last IV cyclophosphamide dose (median (IQR))	79 (19–177)
Other concurrent immunosuppressive agents (Number (%))	
- Mycophenolate mofetil	33 (15)
- Azathioprine	17 (8)
- Methotrexate	13 (6)
- Anti-TNF alpha therapy	9 (4)
- Belimumab	11 (5)
- Tocilizumab	4 (2)
Concurrent prednisolone (Number (%))	111 (50)
- Total daily dose (mg) (median (IQR))	2 (0–5)

Autoimmune disease patients were included from Cambridge (*n* = 213) and Leicester (*n* = 10). Data are reported at the time of most recent sampling. ^a^Other includes polyarteritis nodosa, IgG4 disease, IgA vasculitis, glomerulonephritis including focal segmental glomerulonephritis (FSGS) and membranous nephropathy

### Antibody titres by group and vaccine choice

The Pfizer vaccine was administered in 299 patients (43.2%), AZ in 379 (54.8%). Vaccine type was unknown in 14 (2%). Antibody levels were measured at a median 30 days (IQR: 28–37) after first vaccine dose and 30 days (IQR 30–34) after second vaccine dose. The median intervals between the first and second doses of the Pfizer and AZ vaccines were 77 days (IQR 71–78) and 77 days (IQR 70–80) respectively. In the healthy control cohort, all s-antibody titres were measured at 28 days post-second vaccine dose.

**Fig. 1:**
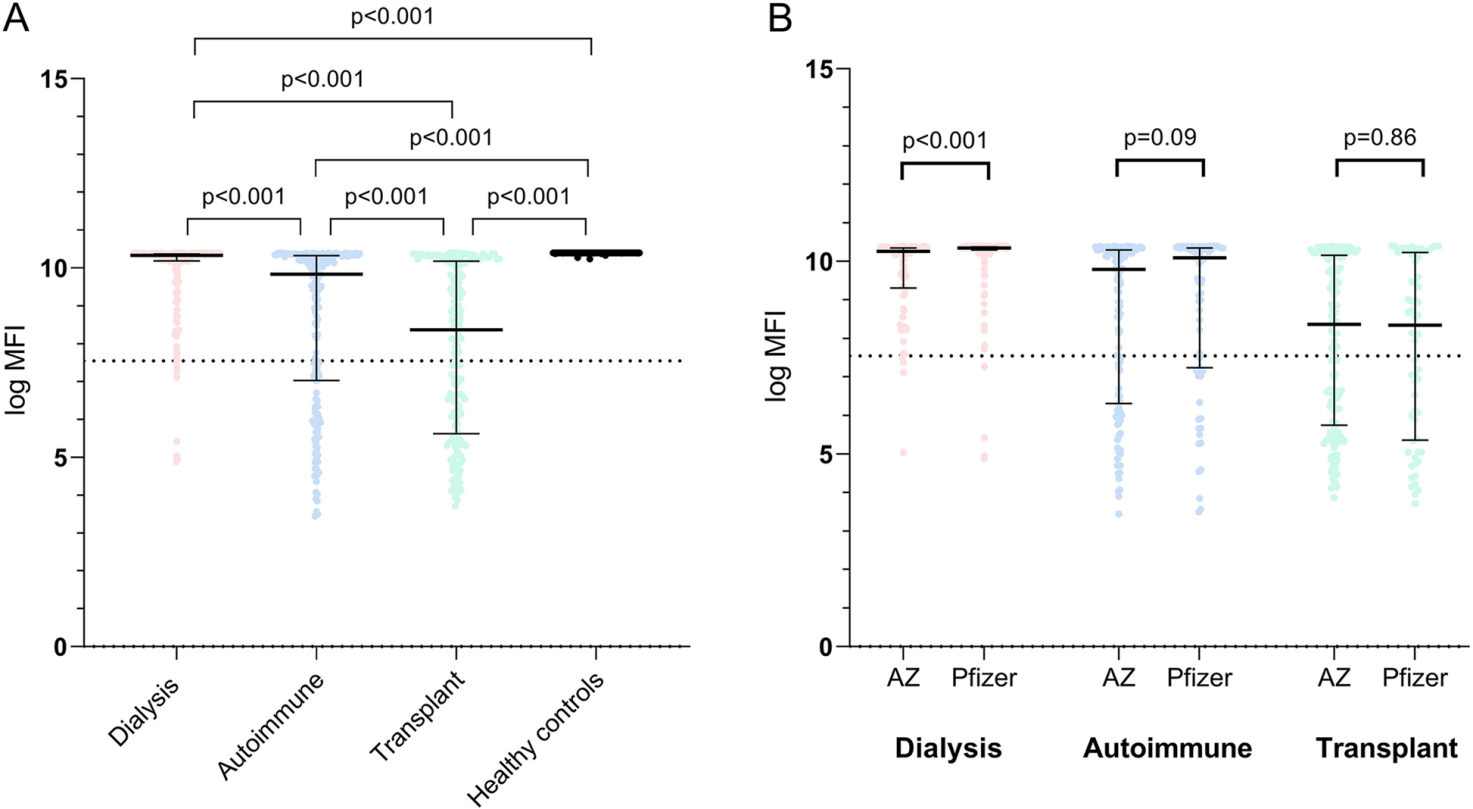
Differences in SARS-CoV-2 antibody responses in three immunocompromised patient populations. **A** Antibody responses following first and second vaccine dose by group. **B** Antibody responses by group and vaccine type. Panels show log MFI of SARS-CoV-2 spike protein IgG antibody responses sampled median x days following first vaccine dose and x days after second dose of vaccine. The positive threshold (IgG antibody titre 1896, log MFI 7.55) is indicated by the horizontal dotted line. The bars represent medians and interquartile ranges and dots represent individual patient results

Median spike protein antibody titres were higher at 28 days-post second vaccine dose after Pfizer vaccine (30,178 [IQR 7800 – 31,813]) compared to the AZ vaccine (14,539 [IQR 794–29,888]) across the whole cohort (not including healthy controls; *p* < 0.001). After second dose of vaccine, median spike protein antibody titre was 30,807 (IQR 26,593 – 31,914) in dialysis, 18,673 (1127 – 30,470) in autoimmune disease and 4285 (295–26,230) in transplant patients (*p* < 0.001 for all between-group comparisons; Fig. 1A). A higher proportion of dialysis patients had a positive antibody response (227/236; 96%) than those with autoimmune disease (140/201; 70%; *p* < 0.001) or a transplant (109/209; 52% *p* < 0.001). Within the dialysis cohort: median s-antibody titres in HD were higher with Pfizer 31,135 (IQR 29,544 – 32,025) compared to 28,619 (IQR 11,086 – 31,263) with AZ (*p* < 0.001). No differences in titre by vaccine type were observed in either the autoimmune (*p* = 0.09) or transplant cohorts (*p* = 0.86) (Fig. 1B).

### Antibody response by MMF and prednisolone status

**Fig. 2:**
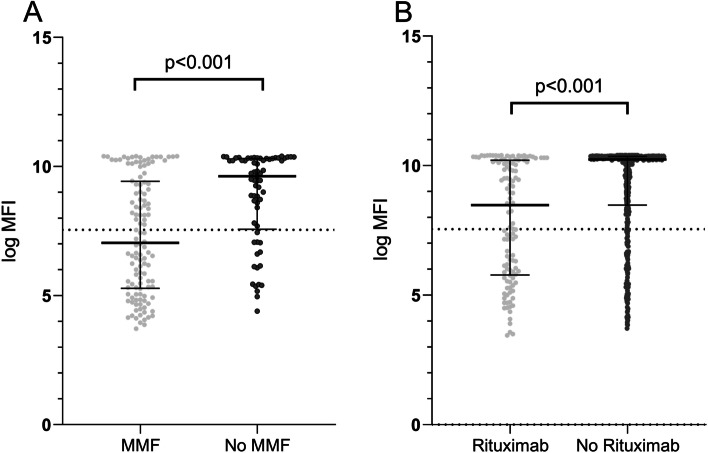
Effect of immunosuppression on SARS-CoV-2 antibody responses. **A** Antibody responses in transplant patients: MMF versus no MMF. **B** Antibody responses in autoimmune patients by prior rituximab exposure. Panels show log MFI of SARS-CoV-2 spike protein IgG antibody responses sampled median x days after second dose of vaccine. The positive threshold (IgG antibody titre 1896, log MFI 7.55) is indicated by the horizontal dotted line. The bars represent medians and interquartile ranges and dots represent individual patient results

Within the transplant cohort, median s-antibody titres were lower in those taking MMF (1145 [IQR 199–12,258]) compared to those not taking MMF (15,091 [2197 – 30,059]; *p* < 0.001; Fig. 2A). Median s-antibody titre at 28 days-post second vaccine dose was lower in those taking prednisolone in any cohort (9593 [IQR 438–29,496]) compared to those not taking prednisolone (29,182 [IQR 6438–31,662]; *p* < 0.001). A higher proportion of those not taking prednisolone had a successful vaccine response (327/391; 84%) than those who were (144/231; 62%; *p* < 0.001).

### Effects of rituximab timing on vaccine response in autoimmune patients

Median s-antibody titres correlated strongly with time from most recent Rituximab treatment (Spearman’s correlation coefficient 0.46; *p* < 0.001), with increasing time from dose correlating with higher s-antibody titres. A lower median s-antibody was seen in those who had received Rituximab in the 6 months preceding a second vaccine dose (690 [IQR 175–12,767] compared to receiving a second vaccine dose greater than 6 months since most recent Rituximab treatment (27,975 (1534 – 31,199); *p* < 0.001; Fig. 2B). No difference was seen in median s-antibody titres between those who received a second vaccine dose < 1 month since previous rituximab treatment compared to 1–3 months since rituximab treatment, and similarly, no difference was seen in those receiving a second dose of vaccine between 1 and 3 months since Rituximab treatment compared to 3–6 months since Rituximab treatment. A lower median s-antibody titre was seen in those having a second dose of vaccine between 3 and 6 months from most recent Rituximab treatment (1341 [IQR 207–12,158]) compared to those receiving a second dose of vaccine 6–12 months since previous Rituximab treatment (10,075 [IQR 553–30,226]; *p* = 0.032). Similarly, those receiving a second dose of vaccine between 6 and 12 months since most recent Rituximab treatment had a lower median s-antibody titre than if rituximab had been given more than 12 months prior to second dose of vaccine (30,648 [IQR 27975–32,114]; *p* = 0.0035).

### Multivariate regression of factors associated with odds of vaccine response

**Fig. 3:**
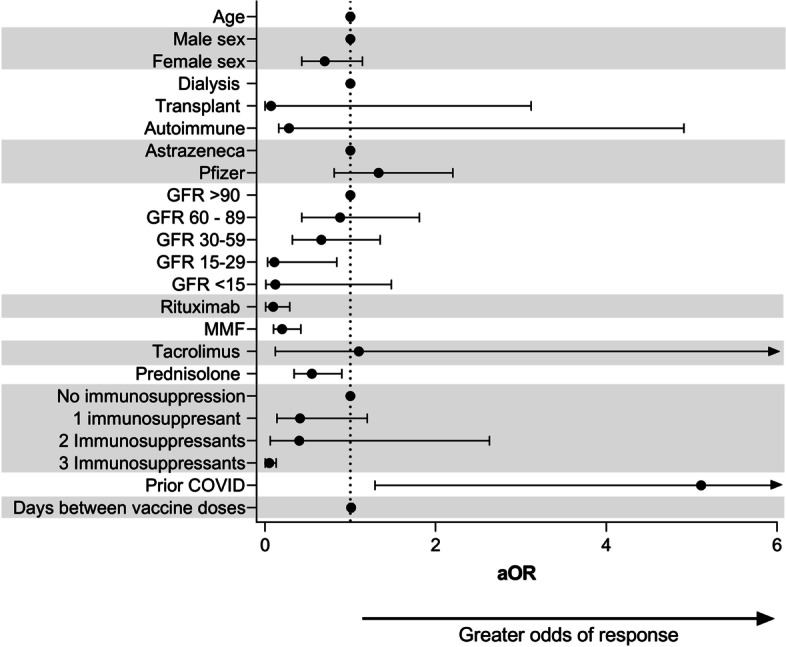
Predictors of antibody responses following vaccination. Forest plot and adjusted odds ratio table from a multivariate logistic regression model including antibody response, patient group, vaccine choice, age, sex, MMF status and Rituximab status

In a multivariate logistic regression model, lower odds of vaccine response were associated with triple immunosuppression (adjusted odds ratio [aOR] 0.016; 95% CI 0.002–0.13; *p* < 0.001), MMF in transplant patients (aOR 0.2; 95% CI 0.1–0.42; *p* < 0.001), prior rituximab within the last 12 months for autoimmune disease (aOR 0.29; 95% CI 0.008–0.096; *p* < 0.001) and GFR less than 29 ml/min in patients receiving immunosuppression (aOR 0.031; 95% CI 0.11–0.84; *p* = 0.021 (Fig. 3). Concurrent prednisolone in any group was associated with a lower odds of a successful vaccine response (aOR 0.55; 95% CI 0.34–0.90; *p* = 0.017). Prior PCR confirmed Covid-19 infection was the only factor associated with a higher odds ratio of vaccine response (aOR 5.11, 95% CI 1.29–20.35; *p* = 0.021).

#### Breakthrough infections

PCR confirmed infections at greater than 28 days post-vaccination were reported in 56 patients to date; 23 dialysis patients (9%), 17 autoimmune disease patients (8%) and 16 transplant patients (8%). Of these breakthrough infections, 31 were male and 25 were female. Post-vaccination median spike antibody titres were lower in those who had breakthrough infections compared to those who did not (11,511 [IQR 1215 – 29,506] vs 30,036 [IQR 6110 – 32,459]; *p* = 0.0001). A ROC analysis identified a cut-off for spike antibody titre of 7629 for identifying the group who had breakthrough infection, with an AUC of 0.44, however sensitivity and specificity were low at 61 and 27% respectively. There was a trend towards lower age in those with breakthrough infections (mean 57 years [SD 15.7] in those with breakthrough infection vs 61 years [SD 18] in those without; *p* = 0.08). There was no difference in vaccine type in those with breakthrough infections (29 had AZ, 27 had Pfizer; *p* = 0.73).

## Discussion

Nephrologists are involved in the care of a heterogeneous group of complex patients. These individuals often have comorbidities other than their kidney diagnosis, and are vulnerable to severe Covid-19 infection. This multi-centre study provides real world comparable data on vaccine efficacy in three immunocompromised renal populations compared to healthy controls. More than 50% of patients in this cohort had received the AZ SARS-CoV-2 vaccine, enabling comparison between mRNA and adenoviral vaccines to be made.

In all patient groups, antibody titre and the proportion of patients with positive antibody responses incremented following a second dose of vaccine. Although positive antibody responses were observed in 97% of dialysis patients, only 52% of renal transplant recipients and 70% of autoimmune patients had positive titres after the second vaccine dose. Median titres in transplant recipients were lower than both autoimmune and dialysis patients, and median titres in autoimmune patients lower than dialysis patients. In dialysis patients, antibody responses were higher with the mRNA Pfizer vaccine compared to AZ adenovirus vaccine and lower overall compared to healthy controls. A lower spike antibody titre following 2 doses of vaccine was associated with a higher likelihood of breakthrough infection.

Key predictors of poor vaccine response were receiving triple immunosuppression or concurrent MMF in transplant recipients, immunosuppression use in patients with CKD stage 4/5 and B cell depletion (prior rituximab therapy) in autoimmune patients. There was a clear positive correlation between time since last rituximab and antibody titre with 94% of patients achieving a positive antibody response when a second vaccine dose was administered more than 12 months after last rituximab dose. These data suggest that repeating vaccination more than 12 months after last Rituximab dose may have a beneficial effect on antibody response to vaccination. For stable patients receiving ongoing repeat rituximab dosing, where clinically appropriate, postponing routine rituximab dosing to optimise vaccine timing and response may be considered, provide close monitoring of patients is in place to avoid relapse [19]. The majority of patients in our autoimmune disease population received rituximab for ANCA-associated vasculitis (AAV), where for remission maintenance fixed interval 6 monthly rituximab dosing is frequently used [20]. Alternative dosing strategies with longer dosing intervals and dosing according to peripheral blood CD19 B cell return and PR3/MPO-ANCA rise may be reasonable, temporarily, in stable vasculitis patients with low relapse risk and experienced clinician oversight, to help optimise vaccine responses [19].

Strengths of this study are the inclusion of three renal patient populations and healthy controls, with evaluation of antibody response on the same diagnostic platform enabling results to be directly compared. In addition, the large number of rituximab treated patients is of particular interest and has enabled the time dependent effect of rituximab to be demonstrated on vaccine response. The frequency of prior COVID 19 infection was low in our population which allowed assessment of vaccine response without the confounding effect of prior infection in the majority. Limitations of the data presented include lack of data on T cell response and neutralising capacity of the antibodies. This is particularly important for those individuals with sub-optimal antibody titres, in whom a weak positive result may offer a false reassurance/overestimate of protection and a borderline negative antibody result may falsely imply no protection at all. Work is ongoing to assess the durability of response and the impact of third dose vaccinations in these immunocompromised renal patient populations.

## Conclusion

These data provide insight into patient subgroups at greatest risk for suboptimal SARS-CoV-2 vaccine response. Although, it is not possible to make individual patient recommendations based on this data, patients on triple immunosuppression or MMF following renal transplantation, autoimmune patients receiving rituximab, and patients with CKD stage 4/5 receiving immunosuppression are at greatest risk for poor vaccine response. These groups should be prioritised for repeated vaccination. At the time of vaccination, temporary reduction of immunosuppression could be considered, but a careful balance between transplant rejection risk/autoimmune disease flare and achieving adequate vaccine response is required, and decisions should be made on an individual basis only by an experienced nephrologist. Reducing prednisolone dose alone, where possible, may have a beneficial effect on response to vaccination. For autoimmune patients, the positive correlation between vaccine response and time since rituximab suggests that delaying routine remission maintenance dosing could be considered; provided relapse risk is deemed to be low and adequate relapse monitoring is in place. Although third and fourth SARS-CoV-2 vaccine doses may allow a further increment in the proportion of patients with detectable antibodies, it remains vital that antibody durability and function is assessed in immunosuppressed patients to identify the most vulnerable sub-populations in whom additional COVID-19 prevention strategies will be of greatest benefit.

## Data Availability

The datasets generated and/or analysed during the current study are not publicly available as the study is ongoing with further time points for data collection, and final analysis of the dataset is currently incomplete, but are available from the corresponding author on reasonable request.
